# "All Sorts and Conditions" of Women

**Published:** 1889-01-12

**Authors:** 


### The Doctor's Pulpit.

### "ALL SORTS AND CONDITIONS" OF WOMEN.

### Four "Created" Women.

Whether it is more scientifically accurate to call characters in fiction "creations" or "evolutions," who shall decide? The use of language is so infinitely varied that it would be impossible to obtain anything like a universal agreement as to the precise meaning of either of the terms. Practical people, happily, do not care to waste time in splitting hairs, either grammatical or metaphysical, and as there is a pretty general usage which calls the men and women of fiction "creations," and their creators "authors," we may feel ourselves on sure ground in following the "common sense of most." The four "created" women we wish to think and speak about with women readers are quite familiar to every student of novels. They are, Di Vernon, Shirley Keeldar, Mary Thorne, and Catherine Elsmere. Every man has probably been in love with one or more women who existed nowhere else but in the imagination of a novelist. And probably he has several times "changed his mind" and given up an early love in favour of a later. But it is a great happiness to some men to be abandoned polygamists in their novel-reading. They find that it is not at all necessary to be "off with the old love before they are on with the new." The present writer professes a graduated measure of affection for at least a score of "heroines," whilst to the four already mentioned he vows a fidelity, which he thinks "nor time nor circumstance may change."

It is not impossible that some readers will wonder what principle of selection has brought together in the mind four such different and variously-conditioned women as Di Vernon, Shirley, Mary Thorne, and Catherine Elsmere. There is no difficulty about it. Persons who are accustomed to scientific classifications easily learn to distinguish leading characteristics. The one feature common to all those four women was that they were "true as steel." They were not merely "true" to their lovers and husbands, but true to the very core of their innermost selves. Diana Vernon loved her father and her "cause" with a glorious self-abandonment of loyalty. There was nothing she would not have sacrificed for them. The reader of seventeen thought such loyalty strained and unnatural, and would have preferred more decided evidences of a lover's warmth than Diana ever showed in the earlier and middle part of her acquaintance with Frank Osbaldestone. At that early age the boy, if he be a lover, is apt to think that love, in a very special sense, should decidedly be "lord of all." When twice seventeen have been reached and passed, the mind changes. It is then understood that the devoted daughter, the loyal adherent of a venerable, even if fallen cause, is not only in herself worthy of the sincerest admiration, but will surely make the noblest of wives to a manly and chivalrous husband.

Shirley is the kind of woman who appeals to grave and mature Louis Moores all the world over. She was young, but not childish. There was nothing of the mere girl about her. She could not love easily, and when she did love she was not at all proud of the fact, but rather, perhaps, ashamed, or at least impatient and angry. It is quite a commonplace thing to love, and Shirley was not a commonplace woman. In her district of Yorkshire "lasses and lads," mere children, might be seen by the dozen, strolling about the fields and lanes on summer evenings in attitudes suggestive of the most absurd kind of mooning calf-love. When John walks with his arm round Eliza's waist along the public road, and has not the sense to take it away even during the brief moments in which you are walking past the pair, the involuntary thought will rise in your breast, "Can I ever have been in all my lifetime such an utter idiot as that lout looks?" Shirley would have permitted no man to be other than a self-respecting creature, even in the moments of her tenderest love. Her stern womanly dignity very seldom permitted itself to be warmed by the bright and ardent fire which burned within. Like all the noblest women, she felt that love itself was to be the servant and not the master. Shirley might have loved many a man; she could only give up her love to one who, in all the kingly qualities of truth, intelligence, and self-control, was greater even than herself. Such a woman is to be wooed before she is to be won, and when she is won the throne of her heart is a place of which any monarch might be proud. There are not many Shirleys; the more's the pity.

Mary Thorne is every man's choice, as Dr. Thorne, her uncle, is every worthy doctor's hero and model. One always feels that, though Frank Gresham was a very good fellow, he did not deserve Mary and ought not to have been permitted to marry her. When Trollope created her he spurred himself up to do the very best that was in him. He maintained his high level to the very last chapter of *Doctor Thorne* so far as she was concerned; but when he produced Frank he evidently made no effort at all, but allowed himself to drop without reserve into respectable commonplace. Even Frank's father was better than Frank. One would have liked Mary to marry a hero, and to have shown to all Barsetshire the grand stuff she was made of. But Trollope could not have created a hero. He did not believe in heroes, and no man can go in practice beyond the measure of his belief. Was any woman in the world ever so really gentle and lovable, and at the same time so strong and true, as Mary Thorne? Perhaps not. If women were so womanly perfect as the lover's, the poet's and the novelist's imagination paints them, it would perhaps be not so well for us. Our divinities would then be in our homes, and we should not incline to seek one elsewhere. But what married man of forty, with a houseful of children, and a wife whose temper is not always as placid as a summer lake, does not sometimes sigh for "the days that are no more," the days when "Alicia" could no more have used the "acid" which flavours her speech now than she could have presented a revolver at dear Edwin's head? It is a disappointing world, is it not, men and women of forty?

Let us bid goodbye to our "Dear Mary Thorne" for awhile, and speak with and about one of the latest of fiction's creations — Catherine Elsmere. Catherine, though only introduced to the world last year, has already been "analysed" over and over again. It is deeply disappointing to find her so little admired by women. They all prefer Rose. So would most boys, and perhaps girls. But *Robert*

*Elsmere* is not a book for boys and girls; and boys and girls probably have not yet decided that it is really a first-rate novel. It is to men and women we look for judgment on such a book.

Comparing Catherine Elsmere with the three already named, and putting each of the four separately into the crucible which he uses for the purest kind of mental and moral gold, the writer sees her come out first, noblest, most truly and profoundly womanly of all. She possessed in unlimited measure that without which neither man nor woman can rise to the level of the greatest, and, lacking which, woman especially, although she may be a worthy compound of sense and kindly feeling, can never be that beautiful, heavenly, and perfect creature which Almighty God designed that she should be. Man, to be typical, must look into and partake of the infinite. Woman still more should see and live among divine as well as among human things. Catherine Elsmere was one who was of the heaven as well as of the earth. There lay her immovable strength, there her beautiful purity, there her steadfast courage, there her power to triumph over every impossibility which seemed to her to lie across her path. Does any reader say she had a "narrow faith?" Has that reader a "faith" at all? Faith that is worth so much as a name cannot be put on and off like a glove. The man of facile belief is the man of feeble intellect and feathery soul. The writer is as far as possible from holding the almost unmodified Calvinism of Catherine Elsmere's creed; but he is not such an inexperienced infallibilist as to suppose that Catherine's creed was necessarily either false, or narrow, or unintellectual, because it does not agree with his. Catherine's creed satisfied Catherine's father, and even greater men. If intellects be compared, Calvinism can show at least as many powerful minds as the opposite doctrines of ultra-freewill.

But this, of course, is not the place for a theological discussion. The point to be made is that Catherine Elsmere was a woman of powerful mind, trained to a narrow range of thought, and yet guided into such ways of searching into and living in union with the infinite and the perfect that she became a kind of divine woman. Such a woman regenerates what she touches. A world of Catherine Elsmeres would expel the devil from human society. They would make all men new and nobler creatures. They would bring up children who would understand the meaning of truth, honesty, courage, and manliness: who would hate lies, and vanity, and shams, and selfishness. A state consisting of men and women of Catherine Elsmere's type would be such a state as the world has never seen. Women do not admire, do not sympathise with Catherine Elsmere! Therein they confess how feeble their own brains are, how powerless their intellect, how ignoble their heart. Compared with an every-day commonplace woman, Catherine Elsmere was as Ben Lomond, with its sky-reaching summit, verdure-clothed sides, and the beautiful islet and tree-sprinkled loch at its foot, is to a Dutch hill, or a sandbank at the mouth of the Thames.

